# Mentoring programs for medical students - a review of the PubMed literature 2000 - 2008

**DOI:** 10.1186/1472-6920-10-32

**Published:** 2010-04-30

**Authors:** Esther Frei, Martina Stamm, Barbara Buddeberg-Fischer

**Affiliations:** 1 Research Center for Career Development, Zurich University Hospital, Zurich, Switzerland

## Abstract

**Background:**

Although mentoring is acknowledged as a key to successful and satisfying careers in medicine, formal mentoring programs for medical students are lacking in most countries. Within the framework of planning a mentoring program for medical students at Zurich University, an investigation was carried out into what types of programs exist, what the objectives pursued by such programs are, and what effects are reported.

**Methods:**

A PubMed literature search was conducted for 2000 - 2008 using the following keywords or their combinations: mentoring, mentoring program, medical student, mentor, mentee, protégé, mentorship. Although a total of 438 publications were identified, only 25 papers met the selection criteria for structured programs and student mentoring surveys.

**Results:**

The mentoring programs reported in 14 papers aim to provide career counseling, develop professionalism, increase students' interest in research, and support them in their personal growth. There are both one-to-one and group mentorships, established in the first two years of medical school and continuing through graduation. The personal student-faculty relationship is important in that it helps students to feel that they are benefiting from individual advice and encourages them to give more thought to their career choices. Other benefits are an increase in research productivity and improved medical school performance in general. Mentored students also rate their overall well-being as higher. - The 11 surveys address the requirements for being an effective mentor as well as a successful mentee. A mentor should empower and encourage the mentee, be a role model, build a professional network, and assist in the mentee's personal development. A mentee should set agendas, follow through, accept criticism, and be able to assess performance and the benefits derived from the mentoring relationship.

**Conclusion:**

Mentoring is obviously an important career advancement tool for medical students. In Europe, more mentoring programs should be developed, but would need to be rigorously assessed based on evidence of their value in terms of both their impact on the career paths of juniors and their benefit for the mentors. Medical schools could then be monitored with respect to the provision of mentorships as a quality characteristic.

## Background

Mentoring was developed in the USA in the 1970s within large private-sector corporations to support junior staff. Since the 1990s, mentoring programs have been introduced in various medical professions, most frequently in the field of nursing. Formal mentoring programs for medical students and doctors, however, were not developed until the late 1990s [1]. Since then, the term "mentoring" has become widespread. In a number of instances there is no clear distinction made between the terms "tutoring", "coaching", and "mentoring". Many definitions of mentoring are in use. The one most frequently cited in English scientific literature (SCOPME [2]) is *"A process whereby an experienced, highly regarded, empathetic person (the mentor) guides another (usually younger) individual (the mentee) in the development and re-examination of their own ideas, learning, and personal and professional development. The mentor, who often (but not necessarily) works in the same organization or field as the mentee, achieves this by listening or talking in confidence to the mentee." *Garmel [3] describes mentoring as "an insightful process in which the mentor's wisdom is acquired and modified as needed, as well as a process that is supportive and often protective. The successful mentor-mentee relationship therefore requires the active participation of both parties. The mentoring relationship can be structured or loose. It can be a relatively short process or an ongoing one. There can be breaks in the relationship, with its re-establishment at some future time. The mentoring relationship is a dynamic one, evolving over time, during which both parties continually define and redefine their roles. It should be considered a process, not an end result, and the relationship must remain non-competitive."

Unlike coaching or counseling, mentoring is a cost-free career-promotion strategy based on a personal relationship in a professional context. Whereas a tutor, teacher/educator, coach, or supervisor mainly focuses on promoting and supporting a junior's professional skills, a mentor is an active partner in an ongoing relationship who helps a mentee to maximize his or her potential and to reach personal and professional goals [4]. Coates et al. [5] differentiate as follows: An *advisor *is a faculty member who provides assistance in scheduling clinical electives and advice on residency applications; a *role model *is someone a student uses as a positive example of how to approach a career in medicine; a *career mentor *is someone who plays an active role in helping the student in his/her professional and personal development. Mentoring also comprises supporting a mentee in coping with stress and in establishing a satisfying work-life balance [6]. Mentoring is a relational process in which five phases can be distinguished: information on career options, developing career plans, focusing on career goals, realization of career steps, and evaluation of career advancement [7,8].

Although several authors report that mentoring is a key to a successful and satisfying career in medicine [4,9,10], there is a lack of mentoring programs for medical students and doctors in most countries [1]. In a prospective study on career development in young physicians, graduates stated that mentoring in medical school would have helped them to make their decision on specialty training earlier and to adopt a more goal-oriented strategy in planning their careers [11]. As a starting point for planning and implementing a mentoring program for students at Zurich University Medical School, a PubMed literature search was conducted with the aim of investigating the following issues: (1) What types of structured mentoring programs for medical students are reported in scientific medical literature between 2000 - 2008? (2) What are the objectives pursued by these programs? (3) What concrete statements, if any, can be identified regarding the effects of mentoring programs? (4) What additional information is given in scientific literature (2000 - 2008) on different aspects of mentoring for medical students?

## Methods

The search strategy for this paper was set up to identify all scientific papers on mentoring programs for medical students. In order to distinguish between scientific and popular literature and between medicine and other professional fields, we decided to limit the search strategy to papers listed in PubMed for the time period 2000 - 2008. The search strategy included the following steps:

(1) The PubMed online search dated December 2008 was conducted with the following keywords or combinations thereof: *mentoring, mentoring program, medical student, mentor, mentee, protégé, mentorship*.

(2) Using this search strategy, we found a total of 438 articles, the titles and abstracts of which were reviewed. Papers that were easily identifiable as lying outside the scope of this study were excluded (n= 353). The remaining 85 papers were retained for the subsequent stage.

(3) The full versions of these papers were reviewed separately by the first and senior author for final inclusion. All papers were written in English, but this was not a selection criterion. The following inclusion criteria were established: Mentoring is to be aimed at medical students; the aim of the mentoring is to support the professional and personal development of the mentee; the mentor is an experienced medical professional; mentoring is in the form of one-to-one mentoring or group mentoring. Only 25 papers met all of the inclusion criteria.

(4) In the final stage, the full versions of these 25 papers were examined.

For *mentoring programs*, the publication data was compiled according to (a) author, year published and country; (b) goal of the program; (c) mentoring model; (d) participants; (e) program evaluation; (f) effects of the program.

For articles referring to *mentoring for medical students in general*, publications were compiled according to (a) author, year published and country; (b) aims of the article; (c) results; (d) conclusion.

## Results

Of the 25 papers that met the four inclusion criteria established, 14 papers [5,12-24] describe formal mentoring programs for medical students, provide information about the goal of the program, the mentoring model used, participants, the nature of program evaluation, and the effects of the program (Table 1).

**Table 1:** Characteristics of 14 mentoring programs for medical students (listed by year of publication)

Author Year Country	Goal of mentoring program	Mentoring model	Participants	Program evaluation	Effects of the program
Coates et al. [5] 2008 USA	Mentoring as part of a 4^th^-year College program	One-to-one and group mentoring	*Mentees: *4^th^-year medical students *Mentors*: Faculty members of the respective college	Pre-/post telephone interviews with students enrolled in the College program and a random sample of a control group	Higher level of satisfaction on the part of the College intervention group with their access to career mentoring, elective advising for scheduling the 4^th^--year and for the residency application process High level of appreciation of on-going contact with peers and faculty, longitudinal clinical experience and research opportunities

Dorrance et al. [12] 2008 USA	Increasing students' interest in internal medicine	One-to-one mentoring	*Mentees*: 1^st^-and 2^nd^-year medical students *Mentors*: Internal medicine faculty members	Quantitative (pre-/pos- program) and qualitative (post program) data collection	Greater interest in internal medicine as a career; career decisions by counseling; higher scholar productivity measured by presentations, publications and research awards

Kanter et al. [13] 2007 USA	Improving students' experiences in medical humanities; supporting students' research projects	One-to-one mentoring	*Mentees*: 3^rd^- and 4^th^-year medical students *Mentors*: Senior physicians	Questionnaire (quantitative and qualitative data from mentees and mentors)	Increased interest in a career as physician-scientist Improved research skills

Kalet et al. [14] 2007 USA	Mentoring as part of an online Professional Development Portfolio (PDP): Supporting professional growth and development; rewarding achievements outside required curriculum	One-to-one and group mentoring	*Mentees*: 1^st^- up to 4^th^-year medical students *Mentors*: Faculty members	Web-based survey tool for the acquisition of quantitative and qualitative data, independent of the PDP	Enrolled students assessed PDP as useful for: tracking own professional development increasing awareness of professional responsibilities preparing for the mentoring sessions

Zink et al. [15] 2007 USA	Providing students with career information, counseling on career decisions and advising on the residency match process	One-to-one mentoring	*Mentees*: A cohort of medical students over four years *Mentors*: Non-physician class counselors, assistant dean, faculty career advisors	Questionnaire (quantitative data)	Career decisions by counseling Broader insight into different medical fields

Macaulay et al. [16] 2007 USA	Advising, guiding and supporting students in their academic and professional development and extracurricular activities	Group mentoring: One mentor for 30 students Structured and informal sessions	*Mentees*: 1^st^- up to 4^th^-year medical students *Mentors*: Senior physicians (faculty members), part-time job	Online questionnaire survey (quantitative data)	Career decisions by counseling Improved networking Increased social support Reduced stress experience

Kosoko-Lasaki et al. [17] 2006 USA	To provide career counseling and group support for underrepresented medical students	Group- and one-to-one mentoring	*Mentees, Mentors*: younger students mentored by advanced students; advanced students mentored by postgraduate students and faculty members	Questionnaire survey (quantitative data)	Improved skills for coping with the demands of higher education Increased social support Facilitated choice of residency program Fostered professional development

Zier et al. [18] 2006 USA	To increase interest in an academic career by providing opportunities to work on research programs	One-to-one mentoring	*Mentees*: 1^st^- to 4^th^-year medical students *Mentors: *Physicians from clinical and science departments	Questionnaire survey (quantitative data)	Increased research skills Increased number of research papers Higher number of postgraduates obtain positions with a research component

Goldstein et al. [19] 2005 USA	Continuous monitoring of the student's progress in medical school	Small group and one-to-one mentoring	*Mentees*: A cohort of medical students over four years *Mentors*: Senior physicians (faculty members)	Results of Mini-Clinical Evaluation Exercise (CEX) and of Objective Structured Clinical Examination (OSCE); students' Portfolio of written work	Improved bedside skills Improved learning skills Evolved ability to monitor the own developmental progress

Coates et al. [20] 2004 USA	Providing students with specialty-specific (Emergency Medicine, EM) career guidance: advice for scheduling their senior year, information about residency programs Role modeling for those embarking on a career path in EM	Two-tier virtual advisor program: First tier: general answers to 14 frequently asked questions (on the Web site) Second tier: Linking students to individual mentors	*Mentees: *Medical students interested in EM *Mentors: *Faculty members with experience in medical education, in advising students and with involvement in a EM residency program	Qualitative email-survey of mentees and mentors	Improved career counseling for a broad range of medical students interested in EM Although written guidelines are given, formal training of mentors is required

Scheckler et al. [21] 2004 USA	Providing an opportunity for continuous professional and personal advice and providing a role model	Group and one-to-one mentoring	*Mentees*: 1^st^- up to 4^th^-year medical students *Mentors*: Experienced physicians (faculty members)	No systematic evaluation, collection of qualitative statements	Broader educational experience Feeling of being psychologically supported Increased awareness of possibilities for integration of professional and extraprofessional concerns

Kalet et al. [22] 2002 USA	Fostering the professional development of the students	Small group mentoring	*Mentees*: 1^st^- and 2^nd^-year medical students *Mentors*: Medical faculty members	Questionnaire survey (quantitative data), focus groups (qualitative data)	Improved professional behavior Development of a professional identity

Murr et al. [23] 2002 USA	Fostering the professional and personal growth and well-being of students	Small group- and one-to-one mentoring	*Mentees*: 1^st^- up to 4^th^-year medical students *Mentors*: Senior physicians	No systematic evaluation	Increased social support Career decisions based on counseling Increased networking

Tekian et al. [24] 2001 USA	To reduce the number of academic difficulties experienced by under-represented medical minority students	One-to-one mentoring	*Mentees*: Minority medical students over four years *Mentors: *Physicians, teachers, advisors, medical students' families, clergy	Personal interviews	Physician mentor: improved medical school performance Other mentors: non-specific personal and professional benefits

Eleven papers [1,3,25-33] refer to mentoring for medical students in general, as well as its significance and impact as far as the students' professional development and success are concerned. These papers are mainly surveys and reports on personal mentoring experiences, while two papers [1,27] are systematic reviews (Table 2).

**Table 2:** Characteristics of 11 mentoring-related studies for medical students (listed by year of publication)

Author Year Country	Aim of the Article	Results	Conclusion
Keyser et al. [25] 2008 USA	Overview: Key domains of research mentorship	1. Mentor selection criteria: -experience and contacts in the mentee's area of research interest 2. Incentives for motivating faculty mentors: - institutional recognition, element for career promotion, awards and time	Research mentorship is a vital part of academic medical education. By establishing mentoring programs, institutions enhance the professional development of future researchers
		3. Factors facilitating the mentor-mentee relationship:	
		- formal matching program, written guidelines for mentors and mentees	
		4. Mentor responsibilities for strengthening the mentee's research abilities:	
		- to provide useful feedback, to supervise the mentees' research	
		5. Mentoring helps mentee	
		- to build a professional network, to apply successfully for grants, to publish manuscripts, to shape personal performance	
		6. Mentor's benefits:	
		- personal satisfaction, increased professional recognition	

Taherian et al. [26] 2008 UK	Overview: Advantages and disadvantages of mentoring	Advantages: - for mentees: shaping of personality, sharing experiences, networking - for mentors: satisfaction, sharing experiences, learning with juniors	Mentoring is a relationship rather than just a set of activities. It is a developmental process for both parties and, if well conducted, represents an enormous benefit
		- for the organization: improvements in doctors' training and satisfaction	
		Disadvantages of mentoring:	
		- conflict of interests between the mentoring and supervising role of the mentor	
		- patronizing attitude of mentors	
		- mentor proposing solutions instead of enabling mentees to find their own way	

Buddeberg-Fischer [1] 2006 Switzerland	Systematic review: Formal mentoring programs for medical students	Types of structured mentoring programs: - peer, group and individual mentoring Short- and long-term goals of mentoring programs: - to stimulate students' interest in a certain medical specialty - training and cooperation in research - to provide career counseling, networking	Formal mentoring programs are of great importance in terms of career support and promotion of junior physicians In the interests of clearly identifying the advantages and disadvantages of formal mentoring, there is a need for a better evaluation
		Short- and long-term effects:	
		- improvement in mentee's professional development and social skills	
		- increased desire to pursue a scientific career	

Buddeberg-Fischer [1] 2006 Switzerland	Systematic review: Formal mentoring programs for medical students	Types of structured mentoring programs: - improvement in mentee's professional development and social skills - increased desire to pursue a scientific career	Formal mentoring programs are of great importance in terms of career support and promotion of junior physicians In the interests of clearly identifying the advantages and disadvantages of formal mentoring, there is a need for a better evaluation

Sambunjak et al. [27] 2006 Croatia and USA	Systematic review: Mentoring in academic medicine: evidence on the prevalence of mentorship and its relationship to career development	Three papers [31,34,35] (two programs) refer to mentoring for medical students: - prevalence of mentorship in academic and health institutions reported in one paper: 36% of 3^rd^- and 4^th^-year medical students - impact of mentorship on personal development, career guidance, specialty and academic career choice, research productivity and success: reported by 60 to 98% of the mentees	Weak evidence to support the perception that mentoring is important for career success

Hauer et al. [28] 2005 USA	Survey: Focus groups of 4^th^-year students with and without mentors Expectations towards mentors, perceived barriers to finding a mentor and suggestions for improving mentoring	Expectations towards a mentor: - devoted to develop a mentoring relationship, friendship and personalized guidance - impact on career development Barriers to finding a mentor: - faculty members seem to be busy, students were put off making an appointment - mentees' career indecision - courses of short duration making it difficult to establish a mentoring relationship Suggestions for enhancement of mentoring: - foster the awareness of the importance of mentorship	Medical students have a desire for supportive, personal and trusting relationships with faculty members, independent of specialty choice

Rose et al. [29] 2005 USA	Overview: Informal mentoring between faculty and medical student Advice on how to be an effective mentor	90% - 95% of students rate mentoring as important; one-third of students report having a mentor Requirements for being an effective mentor: - to be available, to invest in the mentee's personal and professional development, to share experiences, to review the student's progress	Faculty members should be receptive to students' requests for mentoring and provide support when the mentee-mentor-relationship seems appropriate
		Requirements for being a successful mentee: - follow through, accept challenge, set agendas, accept critique	

Cochran et al. [30] 2004 USA	Survey: To identify desirable qualities for surgical role models	Frequency of surgeon mentors: -84% of 3^rd^-year medical students have at least one surgeon mentor Types of surgeon mentors: -Attending surgeons (role of a teacher); -resident surgeons (role of a colleague)	Role models play a substantial part in the selection of a specialty

Garmel et al. [3] 2004 USA	Overview: Requirements for successful mentoring and possible pitfalls	Mentor's qualities and responsibilities: - is non-judgmental and accepts of personal differences - commits time and energy on a regular and ongoing basis - assists in the mentee's identity development - gives honest feedback in a constructive and caring manner	Mentoring is beneficial for both mentees and mentors Students' experience of mentoring in students may encourage them to be mentors themselves in the future
		Benefits for the mentor:	
		- rekindled passion and excitement about the specialty	
		Topics for mentoring:	
		- career choice - application process for residency - academic advancement - career satisfaction - work-life-balance	
		Pitfalls:	
		- inappropriate expectations - breaching confidentiality	

Aagard et al. [31] 2003 USA	Survey: Prevalence and characteristics of informal mentoring relationships among 3^rd^- and 4^th^-year medical students	Prevalence: - 26% of 3^rd^-year and 45% of 4^th^-year students have mentors - no gender difference in the frequency of mentoring relationships Development of mentoring relationship:	Advisors should refer students to potential mentors in the student's field of interest early in medical school
		- 28% during inpatient clerkships	
		- 19% through research activities	
		- 23% by actively seeking on the basis of similar interests	
		Mentoring effects:	
		- Choosing more often a research or an academic career	
		- higher overall satisfaction in medical school	

Hill et al. [32] 2002 USA	Personal perception of mentoring	Mentor's responsibility: - Supporting, counseling, sharing information, being available	Mentorship is a source of fulfillment for the mentor
		Mentee's responsibility: - Seeking the mentor's advice, recognizing limitations of a mentorship	The mentee acquires new perspectives and is led towards his/her goal

Mahayosnand [33] 2000 USA	Short report on a Public Health E-Mentoring program	- Web-based application stating matching criteria - Matching on a central, national database all the year round - Providing essential mentoring literature on the Web site - Over 50% of communications conducted via e-mail	Time- and cost-efficient, but some funding necessary

### Mentoring programs for medical students

All 14 papers [5,12-24] reporting on mentoring programs for medical students between 2000 - 2008 originate in the USA.

#### Goals of the mentoring programs

The mentoring programs reported pursued *different main goals*: (1) to provide career counseling [5,15-17,21,24], (2) to develop professionalism and to support students in their personal growth [14,19,22,23], (3) to increase interest in research and to support an academic career [5,13,18], and (4) to foster students' interest in a specialty for which a future shortage is projected [12,20].

##### Career counseling

Coates et al. [5] report on the College Program at the University of California, Los Angeles (UCLA) for fourth-year medical students. This program has a broad scope, aiming to improve the fourth-year medical school curriculum and provide adequate access to career counseling by faculty mentors. Zink et al. [15] describe a four-phase career development program (CDP) consisting of career-exploring experience, a decision-making phase, preparing the residency application, and interviewing. Students meet with deans and counselors. Macaulay et al. [16] report on a formal Advisory Dean Program (ADP) providing personalized mentoring and advice for each student in terms of career counseling, professionalism, humanism and personal resources. Scheckler et al. [21] from the University of Wisconsin Medical School present their Class Mentor Program (CMP), in which a single mentor is allocated to each class of incoming students and supports the class with clinical and personal advice throughout the four years, up to and including graduation. Kosoko-Lasaki et al. [17] describe the Health Sciences Multicultural and Community Affairs (HS-MACA) Program, a pipeline program targeting students from high school through graduate school which offers special career counseling and mentoring for disadvantaged students (such as female, minority or financially disadvantaged students). Younger students are paired one-to-one with older, more experienced students, and senior students with faculty members. The mentoring program reported by Tekian et al. [24] aims at underrepresented minority students with a view to improving their performance in medical school.

##### Developing professionalism and personal growth

In the online Professional Development Portfolio Program (PDP) described by Kalet et al. [14], mentoring is an integral part of the students' evaluation process in terms of professionalism and career development. The portfolio aims to make students aware of the importance of developing their professionalism; it also supports the setting of goals for the following years in the mentoring sessions. The program published by Goldstein et al. [19] focuses on ongoing personal faculty contact consisting of individual one-to-one mentorship of each student by a faculty member, with an emphasis on bedside teaching and role modeling to enhance clinical skills and professionalism. The same focus is described in the Master Scholars Program (MSP) by Kalet et al. [22], although here, a group of students is mentored by one or two faculty members. The University of California, San Francisco (UCSF) mentoring program, as reported by Murr et al. [23], is moving in the same direction, establishing an advisory college to promote the professional and personal growth and well-being of its students.

##### Increasing interest in research and academic careers

Kanter et al. [13] report on a faculty mentoring program called Scholarly Project (SP), which forms part of a broader program supporting students in their personal and professional development. SP is based on a longitudinal mentoring experience in which the student engages in a hypothesis-driven research project. Each student pursues a focused question in depth with close guidance from a faculty member. SP focuses on the research process, with special attention being paid to ethical issues, and is based on the philosophy that students who become independent, creative thinkers will be better physicians. Moreover, it is believed that if students play an active role in the discovery process, a greater number of them are likely to pursue careers as physician-scientists and, more generally, in academic medicine. Rapid advances in biomedical research call for a large number of physicians being drawn to careers that include a research component. Zier et al. [18] report on a Medical Student Research Program extending over a 10-year period which aims to provide attractive research opportunities including faculty mentoring, acknowledgement of participation, and rewards for achievement to encourage student participation.

##### Fostering interest in certain specialties

The program reported by Dorrance et al. [12] aimed to increase students' interest in pursuing a career as an internist in primary care settings. The faculty launched a medical-student research initiative to increase interest in research during undergraduate medical education. Integrating undergraduate students into internal-medicine research programs and encouraging mentoring relationships with internists working in the primary care field not only produced higher research productivity, but also contributed to a higher percentage of graduates opting for internal medicine training. A similar goal is being pursued by the American Society of Emergency Medicine (EM), which provides a specialty-specific two-tier online career guidance program to attract students to EM and to provide role models for those who choose EM [20].

#### Mentoring models

Six of the programs offer one-to-one mentorships [12,13,15,18,20,24]; in two programs, small groups of students are mentored by a faculty member or a senior physician [16,22], and six programs feature both settings, i.e. one-to-one and group mentoring [5,14,17,19,21,23]. Most mentorships are established in the first two years of medical school and continue up to graduation. In two programs [5,13] in which mentoring forms part of a broader curriculum reform, the mentoring relationship is deliberately not implemented until the fourth year. In some programs the mentors are special faculty career advisors. A virtual mentoring relationship was provided in one program only [20].

#### Effects of the mentoring programs

Eight programs were evaluated by means of questionnaire surveys [12-18,22]; some of these presenting quantitative and qualitative data [12-14,22], others providing only qualitative statements [5,19-21,24]. One program was not evaluated. The UCLA College Program [5] was the only program evaluated by means of a randomized controlled study design (pre- and post-intervention cohorts). The outcome showed that the majority of enrolled students were more satisfied in terms of access to career mentoring, elective advice for scheduling the senior year, and the residency application process; they valued the ongoing contact with faculty members and experienced better research opportunities than students graduating before the program was implemented. All programs reviewed aimed to establish a personal student-faculty relationship, and this was greatly appreciated by the students, especially in ongoing mentoring relationships. The mentors served as role models and contributed to the improvement of professionalism and performance in their mentees [5,12,14,17,19,21,22,24]. The mentored students receiving ongoing career advice and counseling were able to give more thought to the decision on their career, and how this could be matched to their interests and abilities [12,15-17,20,23]. Significant effects were identified in terms of improved medical school performance, increased interest in research, research productivity, and aspiration to an academic career. This was mainly due to the integration of medical students into research collaborations [5,13,18]. The students involved in mentoring programs also felt better supported at a personal level and rated their overall well-being as higher [16,17,21,23,24]. Only Tekian et al. [24] allude to the benefits that a mentor experiences from mentoring students, however.

### Overviews of mentoring for medical students

The literature search revealed 11 papers reporting on mentoring for medical students in general: Keyser et al. [25] provide a conceptual analysis of mentorships, while other authors [3,26,29,32] list tips on how to be an effective mentor and a successful mentee, as well as the advantages and pitfalls of mentoring. The surveys published by Hauer et al. [28] and Cochran et al. [30] report on student attitudes towards mentoring, on the mentoring qualities of mentors, and on the difficulties experienced in finding a mentor. Aagard et al.'s survey [31] gives predictors for having a mentor. In a systematic review, Buddeberg-Fischer et al. [1] report on mentoring models and their effect in the long and short term [1]. Another review, conducted by Sambunjak et al. [27], lists inter alia three papers referring to the mentoring of medical students [31,34,35]. Keyser et al. [25] provide an assessment tool for mentorships. Mahayosnand [33] gives a short report on e-mentoring.

#### Characteristics of a good mentoring relationship

Five of the papers identified reported on the qualities required for being an effective mentor [3,25,26,29,32]. A mentor should be available on a regular and ongoing basis and be non-judgmental, he/she should empower and encourage the mentee, be a role model, build a professional network, and assist in the mentee's personal development. Rose et al. [29] specify the factors involved in becoming a successful mentee, such as the ability to set agendas, follow through, accept criticism, and reassess performance and the benefit of the mentoring relationship. Several authors also point out the difficulties and pitfalls of mentoring [3,26,28]: the short duration of medical school courses, making it difficult for students to make contact with and get to know potential mentors; superiors who make themselves out to be under constant time pressure, thus discouraging students from asking them for mentorship; mentors who put forward solutions instead of enabling mentees to find their own way. Aagard et al. [31] report that the students most likely to find a mentor are those who, having made their choice of career, decide to go in for research. All of the papers conclude that mentoring is an essential part of medical education that enhances the professional and personal development of future physicians and researchers, but only Keyser et al. [25] provide an assessment tool for monitoring institutions in terms of providing mentorships.

## Discussion

Below, important aspects of the papers reviewed are discussed, addressing the issues of appreciation of mentoring, requirements for mentors and mentees, effects of mentoring programs, shortcomings, and suggestions for the design of future mentoring programs.

### Appreciation of mentoring

It is striking that most papers originate in the USA, and few or no reports were searched from other countries using the described criteria and database. Mentoring for medical students is well established in some US medical faculties, and personal and financial resources are available for implementing these programs [5,13,14]. Even more important is the prevalence of the attitude among senior faculty members and faculty authorities that an investment in the juniors' careers is vital in medical education [25,29]. Most authors emphasize that the mentoring relationship is a reciprocal process which supports juniors in their careers; the benefits as far as the mentors are concerned, however, are rarely described [3,25].

Experience of mentoring programs in Switzerland has shown that faculty members and authorities often think that mentoring should be provided for advanced postgraduate trainees only [7], and should focus on research mentorship [11]. Another problem in medical schools in Europe is the high number of students; in Switzerland this number peaks at 220 students per university per year. One way of making mentoring available to all students, however, would be to provide it in groups of up to eight students.

### Requirements for mentors and mentees

Most conceptual and survey papers focus on the qualities required to become an effective mentor [3,25,28,29,32]. A confidential relationship and the mentor's commitment to his/her mentee's professional and personal development are considered to be the main requirements. Unfortunately, it is seldom mentioned whether mentors are assigned or self-appointed. In faculty mentoring programs, all senior faculty members are supposed to mentor one or more graduate students. Some authors suggest that mentors should be encouraged to participate in annual mentorship training programs [25]. Others point out in greater detail the qualities that a mentor should possess [26]. Souba [36] argues that a mentor should '**M**otivate, **E**mpower and Encourage, **N**urture self-confidence, **T**each by example, **O**ffer wise counsel and **R**aise the performance bar'. Only a few authors [3,25] point to the benefit for the mentor in terms of increased professional recognition and accelerated productivity in terms of his/her own research. There is an absence of recommendations in terms of the contribution students can make to being a successful mentee [29,32]. As described in the papers on mentoring programs [5,12-19,21-24], it is preferable that the initiative for establishing mentoring relationships be taken by faculty members, senior physicians, and program leaders, i.e. top-down. However, the responsibility for keeping the mentorship going rests with the mentees, i.e. bottom-up. This perspective is not described. Mentees are required to make themselves out to be proactive juniors. As found in a study on career support in junior academics [11], being proactive and acting on one's own initiative are behaviors by which ambitious and smart students were recognized by faculty members. If juniors prove to be committed, senior staff will approach them to seek their collaboration in research projects. Over time, a reciprocal relationship between juniors and senior staff is established in most cases.

### Effects of mentoring programs

Evidence from the reviewed papers shows that three factors are important for effective mentoring programs. Firstly, for students pursuing an academic career, a one-to-one mentorship with an advanced scientist involving the junior in his/her research proves most effective. Secondly, the mentor must serve as both a professional and personal role model. Thirdly, provision of career counseling by mentors leads to juniors' making an earlier choice in terms of specialty and career.

It has to be said, however, that most of the evaluation studies on the effects of student mentoring programs are not based on validated questionnaires. Consequently, there is only weak evidence that mentoring is important for career success, as pointed out in Sambunjak et al.'s review [27]. Future mentoring programs would benefit from pre-/post-evaluations and randomized studies as reported by Coates et al. [5].

A further problem emerges from the studies reviewed. Some aspects of the mentoring programs mentioned appear to overlap with tutoring, counseling and coaching systems. Moreover, the difference between advisor, role model and career mentor, as described by Coates et al. [5], is not always clear-cut [20]. A further question arises as to whether e-mentoring [20] fulfils the criteria for a mentoring relationship, or whether this type of career support should be considered simply as career advice. In our opinion, e-mentoring lacks the essential requirements for mentoring, i.e. that the mentorship should encompass the mentor's personal commitment to the mentee's personal and professional development and career advancement. It would be difficult for a virtual relationship to cover these aspects of mentoring.

### Shortcomings of the papers reviewed

There is an absence of studies into cost-effectiveness. If we compare the cost of conducting a mentoring program with the benefits to students of earlier career choice, better performance, and higher research productivity, the expense seems to be more than warranted. The mentors do their job without any financial incentives. The costs arising relate to program leaders, the holding of workshops, and some social events.

No data is available in terms of whether mentoring could also help students out of medical school if they are obviously not cut out to be physicians. Admittedly, it must be borne in mind that entrance tests and interviews as well as selective exams in the first year of medical school increase the probability that a majority of students will fit the profile of a medical professional.

Furthermore, the negative effects of mentoring are not reported in the studies. As noted in the Resident Mentoring Program at Zurich University Hospital [37], mentoring may be biased on account of institutional interests. It might lack confidentiality if the mentor is a senior physician in the same department responsible for supervising the resident and awarding their qualification. There should be no hierarchical dependency. In the aforementioned Zurich University program, mentees either choose their mentor on their own initiative or the mentorship is set up by the program leader, based on the main interests of the mentee.

### Suggestions for the design of future mentoring programs

In our view, a useful and feasible model for a student mentoring program could be designed using tiers, as reported by Kosoko-Lasaki et al. [17]: younger students are mentored by advanced students, and advanced students are mentored by faculty members or senior physicians/researchers. Mentoring students calls for enjoyment in educating others as well as the ability to act as a role model and instill enthusiasm for a particular field of medicine or research. Female mentors might be especially important for female students, in that they may provide a role model for combining the demands of a job with family commitments. The program leader is called upon to approach qualified, suitable mentors for matching up with the mentees. It is the program leader's task to seek out and maintain contact with potential mentors.

Compared to our review on formal mentoring programs for medical students and physicians [1], the present paper covers the recent period 2000 - 2008, and focuses both on mentoring programs for medical students and on general overviews of mentoring for medical students. It provides a deeper insight into appreciation of mentoring in different countries, and requirements for mentors and mentees to establish an effective and successful mentoring relationship.

## Conclusion

Mentoring is obviously an important career advancement tool, which would benefit from early implementation at medical school. Mentorships must be goal-oriented and rigorously evaluated in terms of the positive outcomes for mentees as well as for mentors. Once the effects of mentoring are more clearly documented, mentoring will receive more appreciation.

## Competing interests

The authors declare that they have no competing interests.

## Authors' contributions

EF has conducted the PubMed literature search, reviewed the papers and compiled the publication data together with MS. BBF reviewed the papers separately. BBF drafted the manuscript, which was critically revised by the other authors. All authors read and approved the final manuscript.

## Pre-publication history

The pre-publication history for this paper can be accessed here:

http://www.biomedcentral.com/1472-6920/10/32/prepub
